# New Variant of Esophageal Atresia

**DOI:** 10.21699/jns.v6i1.464

**Published:** 2017-01-01

**Authors:** Swapnil Harne, Manish Pathak, Kamal Nayan Rattan

**Affiliations:** 1 Department of General Surgery, R.N.T, Medical College and Maharana Bhupal Government Hospital, Udaipur, Rajasthan, India; 2 Department of Pediatric Surgery, All India Institute of Medical Science, Jodhpur, Rajasthan; 3 Department of Pediatric Surgery, Pt. B.D. Sharma, Post Graduate Institute of Medical Sciences, Rohtak, Haryana

**Keywords:** Esophageal atresia, Tracheoesophageal fistula, Esophageal stenosis

## Abstract

Esophageal atresia with tracheoesophageal fistula (EA/TEF) associated with distal congenital esophageal stenosis (CES) is a well-known entity. We encountered three patients of EA/TEF associated with long and unusual CES.

## CASE SERIES

**Case 1:**

A 2.5 kg full term baby presented to us with history of drooling of saliva and intolerance to feed with choking and coughing at the time of feeds. Inability to pass the red rubber catheter into stomach confirmed the diagnosis of esophageal atresia. The chest and abdominal x-ray revealed red-rubber catheter in upper pouch at vertebral level D4. The chest x-ray also revealed right upper lobe consolidation. There was presence of gas in the abdomen confirming the diagnosis of EA/TEF. The Ultrasound KUB was normal. The echocardiography revealed the presence of small VSD. The blood investigations were normal. Right thoracotomy showed wide TEF which was divided and repaired. The upper esophageal pouch was mobilised and esopago-esophageal anastomosis was started. After the anastomosis of posterior wall, a 5 Fr. infant feeding tube was tried to pass across the anastomosis into the stomach, but the feeding tube couldn’t be passed into the stomach due to the presence of stenosis about 2 cm distal to the proximal end of distal esophageal pouch. Two stay sutures were taken at the narrowed part of esophagus and longitudinal incision was made between the stay sutures to find the lumen but there was no negotiable lumen. It was followed further distally, but no negotiable lumen could be found even till the gastro-esophageal junction. Thus, cervical esophagostomy and gastrostomy were performed. Post-operatively patient developed septicaemia and died.

**Case 2:**

A 2.2 kg full term baby was diagnosed pre-operatively as EA/TEF. On thoracotomy, we had similar intra-operative finding like case 1, and patient underwent esophagostomy and gastrostomy. One year later gastric pull-up was done. The patient developed anastomotic leak which was managed conservatively. Presently patient is accepting feeds orally but has anastomotic stricture which required esophageal dilatation twice.

**Case 3:**

A 2.7 kg full term baby diagnosed preoperatively as EA/TEF. The patient did not have any other associated anomaly. There were similar intra-operative findings like case 1 and 2, based on which patient underwent cervical esophagostomy and gastrostomy. The patient is waiting for esophageal replacement surgery.

In all the above-mentioned cases biopsies taken from esophageal stenosis confirmed histological picture consistent with fibromuscular variant of CES.

## DISCUSSION

Literature has mentioned similar cases of EA with TEF with CES at distal esophageal pouch with some lumen. [1-3] To the best of our knowledge, there are no reports published on EA-TEF with such a long segment of CES with no lumen. This finding did not leave us with any option of esophageal dilation or resection with end to end anastomosis of stenosed segment. The only possible alternative was to plan an esophageal replacement. In recent years, various literatures have proven that occurrence of EA with TEF along with CES is a rather commoner phenomenon than anticipated earlier. Hence, we would like to propose that, even though this variant is a rare occurrence, the presence of associated CES in type C TEF should be included in gross classification of EA and TEF as a separate type G, i.e. “a variant with EA and distal TEF with a segmental CES at distal esophageal pouch.”

## Footnotes

**Source of Support:** Nil

**Conflict of Interest:** None declared
